# A new mouse model of typhoid fever using *Salmonella enterica* serovar Paratyphi C as a surrogate pathogen

**DOI:** 10.1128/mbio.03622-25

**Published:** 2026-01-16

**Authors:** Hoan T. Pham, Masatomo Morita, Kohei Yamazaki, Toshihiro Endo, Satoshi Takayama, Azusa Hiyoshi, Takeshi Haneda, Renée M. Tsolis, Andreas J. Bäumler, Toshio Kodama, Hirotaka Hiyoshi

**Affiliations:** 1 Department of Bacteriology, Institute of Tropical Medicine (NEKKEN), Nagasaki University 196838 , Nagasaki, Japan; 2 Programme for Nurturing Global Leaders in Tropical and Emerging Communicable Diseases, Graduate School of Biomedical Sciences, Nagasaki University 200674 , Nagasaki, Japan; 3 Department of Bacteriology I, National Institute of Infectious Diseases, Tokyo, Japan; 4 Laboratory of Veterinary Public Health, School of Veterinary Medicine, Kitasato University 73467, Towada, Aomori, Japan; 5 Phenovance LLC, Chiba, Japan; 6 Department of Microbiology, School of Pharmacy, Kitasato University 47702, Minato, Tokyo, Japan; 7 Department of Medical Microbiology and Immunology, School of Medicine, University of California at Davis 272916, Davis, California, USA; Massachusetts Institute of Technology, Cambridge, Massachusetts, USA

**Keywords:** *Salmonella*, typhoid, mouse model

## Abstract

*Salmonella enterica* serovar (*S*.) Typhi, the etiological agent of typhoid fever, is strictly human adapted, which presents a significant challenge for studying its pathogenesis in animal models. A common strategy to overcome this limitation is to infect mice with *S*. Typhimurium as a surrogate pathogen. Since *S*. Typhimurium is a non-typhoidal serovar that does not encode the virulence-associated capsular polysaccharide (Vi antigen) of *S*. Typhi, we explored whether the mouse virulent typhoidal *Salmonella* serovar Paratyphi C, which expresses the Vi antigen, would be better suited as a surrogate pathogen to study typhoid fever pathogenesis in the mouse. In contrast to the nontyphoidal serovar Typhimurium, which produced lethal morbidity in C57BL/6 mice within a few days after infection, *S*. Paratyphi C demonstrated prolonged colonization of systemic organs for up to 28 days after infection. Analysis of virulence factors revealed that the Vi antigen was important at very early stages after infection (up to 2 days), whereas the type III secretion system encoded by *Salmonella* pathogenicity island 2 became critical at later stages. Vaccination with purified Vi antigen suppressed *S*. Paratyphi C dissemination. Implantation of a biotelemetry device revealed that *S*. Paratyphi C triggered fever after an incubation period of 3 days, which was reminiscent of the prolonged incubation period of typhoid fever. In conclusion, our findings suggest that the use of *S*. Paratyphi C as a surrogate pathogen provides a mouse model for studying typhoid fever pathogenesis and vaccine development.

**IMPORTANCE:**

The emergence of extensively drug-resistant *Salmonella enterica* serovar (*S*.) Typhi poses a serious threat to public health, but its host restriction to humans poses a challenge for studying pathogenesis and vaccine development in animal models. Here, we used *S*. Paratyphi C, a mouse virulent typhoidal serovar that expresses the virulence-associated Vi capsular polysaccharide, as a surrogate pathogen for studying typhoid fever in a mouse model. Our model recapitulates key features of typhoid fever, including clinical symptoms such as a prolonged incubation period, fever, and splenomegaly. Notably, disseminated infection with *S*. Paratyphi C developed after inoculation by the natural oral route. We demonstrate the utility of this model for studying pathogenesis and vaccination. We conclude that our new mouse model for typhoid fever offers a promising platform for evaluating novel therapeutics and vaccine candidates to address the problem of drug resistance in *S*. Typhi and reduce the global burden of typhoid fever.

## INTRODUCTION

From the standpoint of human disease, *Salmonella* serovars can be subdivided into non-typhoidal serovars, which are associated with gastroenteritis, and typhoidal serovars, which are associated with typhoid fever and paratyphoid fever in humans. Typhoid fever is a systemic infectious disease caused by *Salmonella enterica* serovar (*S*.) Typhi. Fecal-oral transmission of the pathogen from human carriers occurs through ingestion of contaminated water or food (1–3). After a prolonged incubation period (4), the disease manifests with fever, whereas additional symptoms, which are not observed in all patients, include abdominal pain, splenomegaly, and the development of rose spots (5). Complications of typhoid fever include intestinal perforation, encephalopathy, and cardiac encephalitis (6). With antibiotic therapy, the fatality rate of typhoid fever is approximately 1%, resulting in 116.8 thousand annual deaths worldwide (7). However, the emergence of extensively drug-resistant (XDR) *S*. Typhi has raised concerns that the fatality rate could rise above 10% as antimicrobial resistance becomes more widespread (7). The World Health Organization (WHO) includes fluoroquinolone-resistant *S*. Typhi in its high-priority group based on the threat the pathogen poses to public health (8).

Understanding the mechanisms through which pathogens establish infections is crucial for developing novel vaccines and therapeutics. *S*. Typhi virulence factors include two type III secretion systems (T3SSs) encoded by *Salmonella* Pathogenicity Islands (SPI)-1 and -2, which facilitate inflammation in intestinal cells and proliferation within macrophages, respectively (9, 10); the typhoid toxin composed of CdtB, PltA, and PltB, which acts on human cells expressing N-acetylneuraminic acid (Neu5Ac) to arrest them in the G2/M phase (11), fimbriae involved in adhesion to host cells (12), and genes located on other SPIs (SPI-3, SPI-8, SPI-10, and SPI-18) (1, 13). In addition, *S*. Typhi produces a virulence-associated (Vi) capsular polysaccharide, known as the Vi antigen. The *viaB* locus on SPI-7 encodes genes responsible for the regulation (*tviA*), synthesis (*tviBCDE*), and export (*vexABCDE*) of the Vi antigen (14, 15). The Vi antigen is a homopolymer of (1, 4)-2-acetamido-3-O-acetyl-2-deoxy-α-D-galacturonic acid (16) anchored in the outer membrane through a reducing terminal N-acetylhexosamine residue modified with two β-hydroxyl acyl chains (17). The Vi antigen inhibits complement activation on the bacterial surface and reduces the production of complement component 5a (C5a), a potent chemoattractant for neutrophils (18). Furthermore, we demonstrated that the Vi antigen evades the bactericidal activity of neutrophils via reactive oxygen species (ROS) by inhibiting the binding of natural immunoglobulin M (IgM) antibodies, which initiates complement activity (19). However, most of these studies have been conducted primarily through *in vitro* experimentation, and the role the Vi antigen plays during typhoid fever remains incompletely understood. Furthermore, purified Vi antigen is one of the approved *S*. Typhi parenteral vaccines (20), but the mechanism by which anti-Vi antibodies confer protection *in vivo* has not been explored.

Animal models are important tools for analyzing the role of virulence factors and for the development of vaccines and therapeutics (21, 22). However, *S*. Typhi is a human-restricted pathogen that cannot infect small animals and non-human primates (23). Two strategies have been employed to overcome this limitation: (i) the use of a surrogate pathogen that is mouse virulent, and (ii) modification of the mouse immune system to increase susceptibility to *S*. Typhi infection. The first strategy commonly utilizes the non-typhoidal *Salmonella* serovar Typhimurium as a surrogate pathogen (24). Limitations of this model include the genetic differences between *S*. Typhi and *S*. Typhimurium, along with the fact that *S*. Typhimurium primarily causes gastroenteritis in humans. An example of the second strategy is to humanize the mouse immune system to increase susceptibility to *S*. Typhi (25–27). Infection of non-obese diabetic-scid IL2rg(gamma)null mice engrafted with CD34^+^ human hematopoietic stem cells (hu-SRC-SCID) with a high-density *S*. Typhi transposon library identified numerous factors required for infection, including genes involved in Vi antigen synthesis (10, 23). However, humanized mice exhibit a number of limitations, including variability of engraftment, high production costs, immunodeficiency, and the fact that animals succumb to infection within a week, which does not mimic the prolonged incubation period of typhoid fever (25, 26, 28, 29). Additional obstacles can be the requirement for a biosafety level-3 animal facility for work with *S*. Typhi, which is a requirement in Japan and some countries in Europe due to the risk of sporadic laboratory infections (30, 31).

Here, we explored whether the use of a surrogate pathogen to study typhoid fever pathogenesis could be improved by using the typhoidal *Salmonella* serovar Paratyphi C instead of the non-typhoidal serovar Typhimurium. *S*. Paratyphi C causes paratyphoid fever in humans, a disease that is indistinguishable from typhoid fever in its symptoms. Additionally, among the more than 2,500 known *Salmonella* serovars, only three synthesize the Vi antigen, which includes *S*. Typhi, *S*. Paratyphi C, and *S*. Dublin, a pathogen associated with an extraintestinal disease in cattle (32, 33). Here, we explored whether *S*. Paratyphi C can be used as a surrogate pathogen to model typhoid fever in mice.

## RESULTS

### *S*. Paratyphi C strain 13428 exhibits prolonged infection in C57BL/6 mice

**Fig 1:**
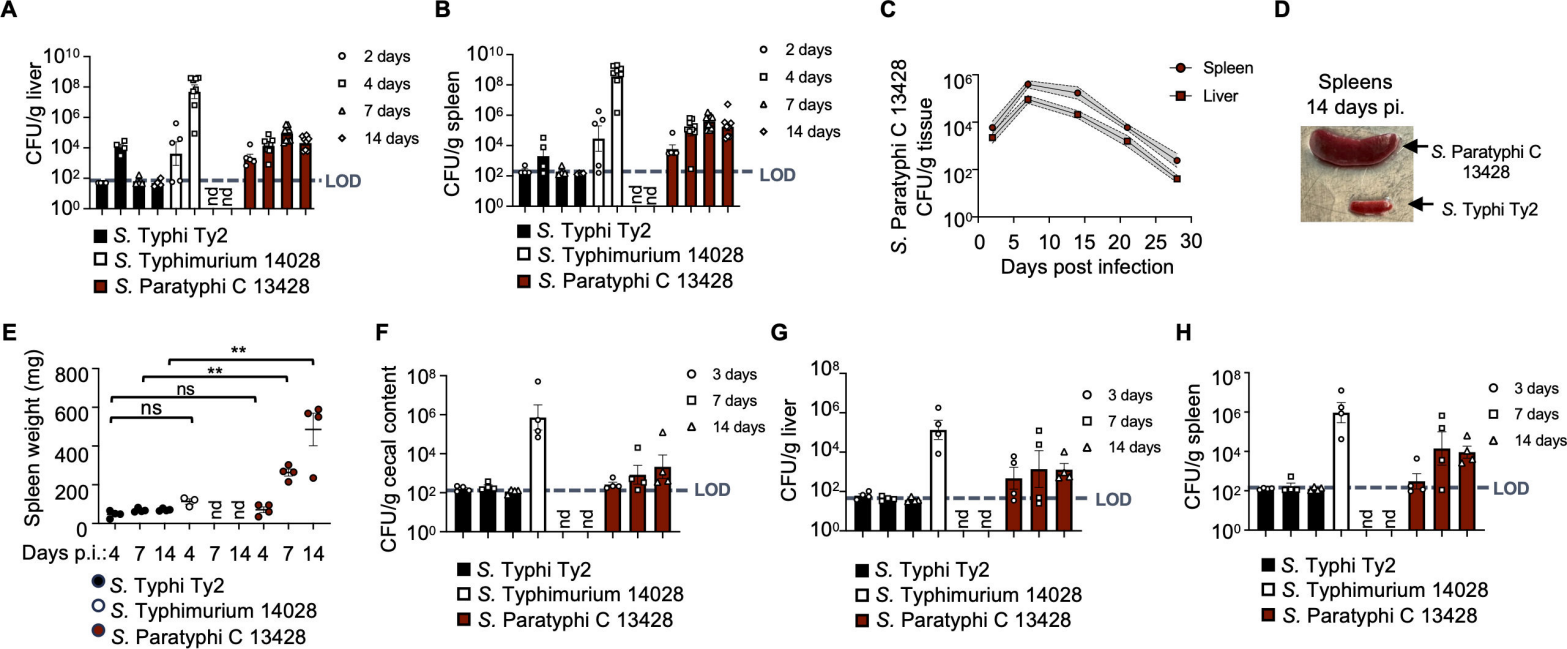
*S*. Paratyphi C 13428 strain exhibits prolonged infection in C57BL/6 mice. (**A–E**) C57BL/6J mice were intraperitoneally infected with 10^4^ CFUs of *S*. Typhi Ty2, *S*. Typhimurium 14028, or *S*. Paratyphi C 13428. The graphs depict the *Salmonella* CFUs recovered from the liver (**A and C**) and spleen (**B and C**) on specific days post-infection (pi.). (**D**) Spleens were collected from C57BL/6J mice infected with *S*. Typhi Ty2 or *S*. Paratyphi C 13428 at 14 days pi.. Splenomegaly was observed in *S*. Paratyphi C infections. (**E**) Spleen collected from mice infected with *S*. Typhi Ty2, *S*. Typhimurium 14028, or *S*. Paratyphi C 13428 on individual days pi. were weighed. (**F–H**) C57BL/6J mice were orally infected with 10^9^ CFUs of *S*. Typhi Ty2, *S*. Typhimurium 14028, or *S*. Paratyphi C 13428. The graphs illustrate the *Salmonella* CFUs recovered from the cecal content (**F**), liver (**G**), and spleen (**H**) on specific days pi. Each symbol represents data from an individual animal. LOD, limit of detection; nd, not determined, as mice did not survive on the days following infection. Bars indicate geometric means ± standard error. Statistical significance is denoted as follows: ns, not significant, defined as *P* > 0.05; **, *P* < 0.01. The analysis was conducted using one-way analysis of variance followed by Tukey’s multiple comparison test for the three groups at 4 days pi. and by Student’s *t*-test for the two groups at 7 and 14 days pi.

In contrast to *S*. Typhi, *S*. Paratyphi C has been shown to infect mice; however, its infectivity has not been thoroughly examined (34–37). Consequently, in this study, mice were challenged with a *S*. Paratyphi C strain 13428, *S*. Typhi strain Ty2, and *S*. Typhimurium strain 14028 using identical conditions to allow for a comparative analysis of their infectivity. We used C57BL/6J inbred mice, a commonly used background for mouse genetics and for studying *S*. Typhimurium infection (38). To establish a murine model of parenteral *Salmonella* infection, 10^4^ colony-forming units (CFUs) of each serotype were injected into the peritoneal cavity of mice, and bacterial counts in the liver and spleen were conducted over a 14-day period. Consistent with previous reports, *S*. Typhi was nearly cleared from liver and spleen within a week after infection, whereas *S*. Typhimurium exhibited rapid proliferation, resulting in the development of lethal morbidity in all infected mice within 7 days (Fig. 1A and B) (26, 39). In contrast to those serovars, *S*. Paratyphi C exhibited slower bacterial proliferation (Fig. 1A and B), resulting in persistence of the pathogen in the liver and spleen of mice for at least 28 days after infection (Fig. 1C). Splenomegaly, a clinical manifestation in 23%–65% typhoid patients (40), was observed in mice infected with *S*. Paratyphi C strain 13428, as evidenced by a significant increase in spleen size (Fig. 1D and E).

One limitation of humanized mice is that although these animals are susceptible to parenteral infection, they are resistant by the natural, oral route of infection (26). To test whether the use of *S*. Paratyphi C as a surrogate pathogen would enable the use of oral infection to study typhoid fever pathogenesis, mice were infected intragastrically with 10^9^ CFUs of *S*. Paratyphi C. *S*. Paratyphi C gradually disseminated into liver and spleen tissues after oral infection and persisted in these organs throughout the experiment (Fig. 1F through H), reminiscent of the gradual onset of typhoid fever (3, 4, 41). In contrast, rapid growth of *S*. Typhimurium in organs resulted in animals becoming moribund by 7 days after infection (Fig. 1F through H). The bacterial load in the organ reached its peak 1 week following oral *S*. Paratyphi C infection, coinciding with the onset of splenomegaly (Fig. 1A through E). This observation prompted us to further investigate the potential of using *S*. Paratyphi C strain 13428 as a surrogate pathogen for studying typhoid fever pathogenesis in the mouse.

### The very-long O-antigen chains expressed by *S*. Paratyphi C diminish the ability of the Vi antigen to evade the neutrophil respiratory burst

**Fig 2:**
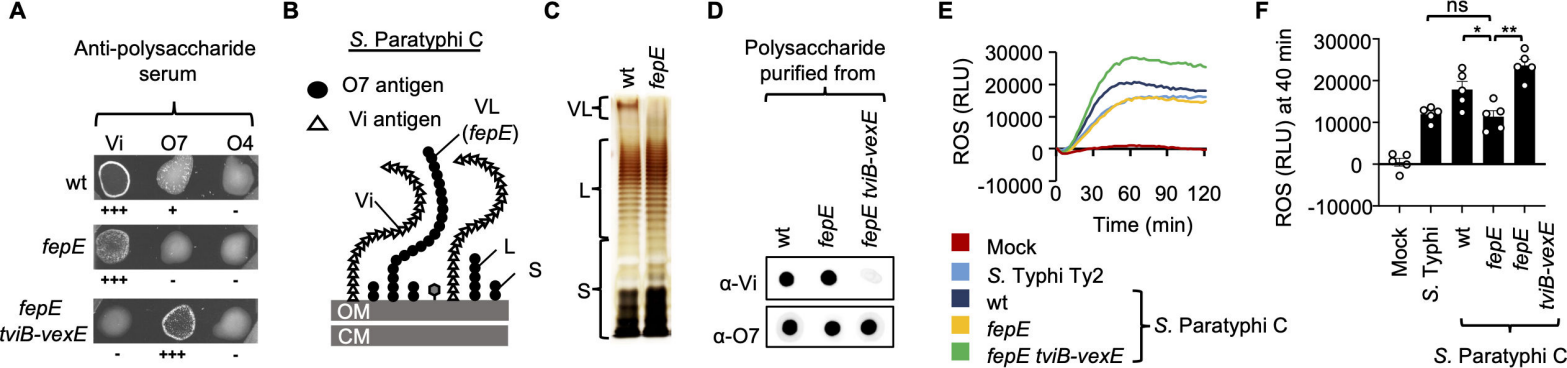
The VL O-antigen chains expressed by *S*. Paratyphi C diminish the ability of the Vi antigen to evade the neutrophil respiratory burst. (**A**) Strains of *S*. Paratyphi C, including wild type (wt), *fepE* mutant, and *fepE tviB-vexE* mutant, were incubated with sera containing anti-Vi, O7, or O4 polysaccharides. The resulting agglutination was categorized as negative (−), positive (+), or robust (+++). (**B**) A diagram illustrating the surface structure of *S*. Paratyphi C, where CM denotes the plasma membrane, L represents long O-antigen chains, OM indicates the outer membrane, S signifies short O-antigen chains, Vi refers to the Vi antigen, and VL denotes very long O-antigen chains. (**C**) LPS purified from the specified *S*. Paratyphi C wt or *fepE* mutant was separated using SDS-PAGE and silver staining. (**D**) Production of the Vi or O7 antigen was detected in polysaccharides purified from the specified strains using rabbit anti-Vi or O7 serum. (**E and F**) Primary neutrophils enriched from murine bone marrow were infected with the specified opsonized bacterial strains, and ROS production was monitored over time using chemiluminescence. (**E**) Representative images illustrating the generation of chemiluminescence over time. (**F**) Quantification of chemiluminescence from five independent experiments measured at 40 min pi. Each symbol corresponds to data from an individual animal. Bars indicate geometric means ± standard error. Statistical significance is denoted as follows: ns, not significant, defined as *P* > 0.05; *, *P* < 0.05; **, *P* < 0.01. The analysis was conducted using one-way analysis of variance followed by Tukey’s multiple comparison test.

*S*. Typhi expresses the Vi antigen that prevents natural IgM from binding to lipopolysaccharide (LPS) O-antigen chains on the bacterial surface, thereby inhibiting slide agglutination with anti-LPS antibodies and the generation of a respiratory burst in neutrophils (19, 42). The *viaB* locus of *S*. Paratyphi C exhibits more than 99% nucleotide sequence identity to that of *S*. Typhi Ty2 (43). However, whereas the Vi antigen blocks slide agglutination of *S*. Typhi (19, 42), antibodies against the *S*. Paratyphi C O-antigen (i.e., rabbit anti- O7 serum) produced a weakly positive reaction during slide agglutination (Fig. 2A) (44). It has been speculated that this difference could be due to reduced synthesis of the Vi antigen in *S*. Paratyphi C (44). However, an alternative explanation is that a weak inhibition of slide agglutination by the Vi antigen of *S*. Paratyphi C is attributable to the presence of very-long (VL) O-antigen chains. LPS of *Salmonella* serovars can be categorized into short, long, and VL O-antigen chains based on the number of O-antigen repeat units that are attached to the LPS core (45). Notably, *S*. Typhi does not produce VL O-antigen chains due to a loss-of-function mutation in *fepE*, the gene encoding the length regulator of VL O-antigen chains (45). Restoration of VL O-antigen synthesis in *S*. Typhi circumvents the Vi antigen-dependent inhibition of natural IgM antibody binding (19). Since *S*. Paratyphi C possesses an intact *fepE* gene, we reasoned that synthesis of VL O-antigen chains might prevent the Vi antigen from effectively blocking slide agglutination (Fig. 2B). Consistent with this idea, slide agglutination with anti-O-antigen serum was eliminated when we inactivated the *fepE* gene in *S*. Paratyphi C (Fig. 2A). Sodium dodecyl sulfate polyacrylamide gel electrophoresis (SDS-PAGE) analysis of LPS extracted from the *S*. Paratyphi C wild type revealed a high-molecular weight band representing VL O-antigen species, which were absent when LPS extracted from an isogenic *fepE* mutant was analyzed (Fig. 2C). Next, we deleted the Vi antigen biosynthesis genes in a *S*. Paratyphi C *fepE* mutant and confirmed a loss of Vi antigen synthesis in the resulting *S*. Paratyphi C *fepE tviB-vexE* mutant using dot blot analysis of polysaccharides extracted from cultured bacteria using anti-Vi antiserum (Fig. 2D). Deletion of the Vi antigen biosynthesis genes resulted in robust slide agglutination with anti O-antigen serum (Fig. 2A), suggesting that the Vi antigen efficiently blocks antibody binding to short and long O-antigen chains but not VL chains.

By blocking the binding of natural IgM, *S*. Typhi reduces the generation of complement fragment C5a and inhibits activation of the complement cascade, thereby attenuating the neutrophil respiratory burst (19). Therefore, we measured the amount of ROS by chemiluminescence to determine whether the *S*. Paratyphi C Vi antigen has a similar activity. ROS production elicited by the *S*. Paratyphi C wild type in primary murine neutrophils was elevated compared to the response elicited by *S*. Typhi. An *S*. Paratyphi C *fepE* mutant elicited ROS production at similar levels as *S*. Typhi, whereas deletion of the Vi antigen biosynthesis genes (*S*. Paratyphi C *fepE tviB-vexE* mutant) resulted in a marked increase in ROS production compared to the other strains (Fig. 2E and F). These data suggested that an *S*. Paratyphi C *fepE* mutant was best suited for use as a surrogate pathogen to study the role of the *S*. Typhi Vi capsular antigen during infection of mice.

### The Vi antigen enhances recovery of *S*. Paratyphi C from organs during the early stage of systemic infection

**Fig 3:**
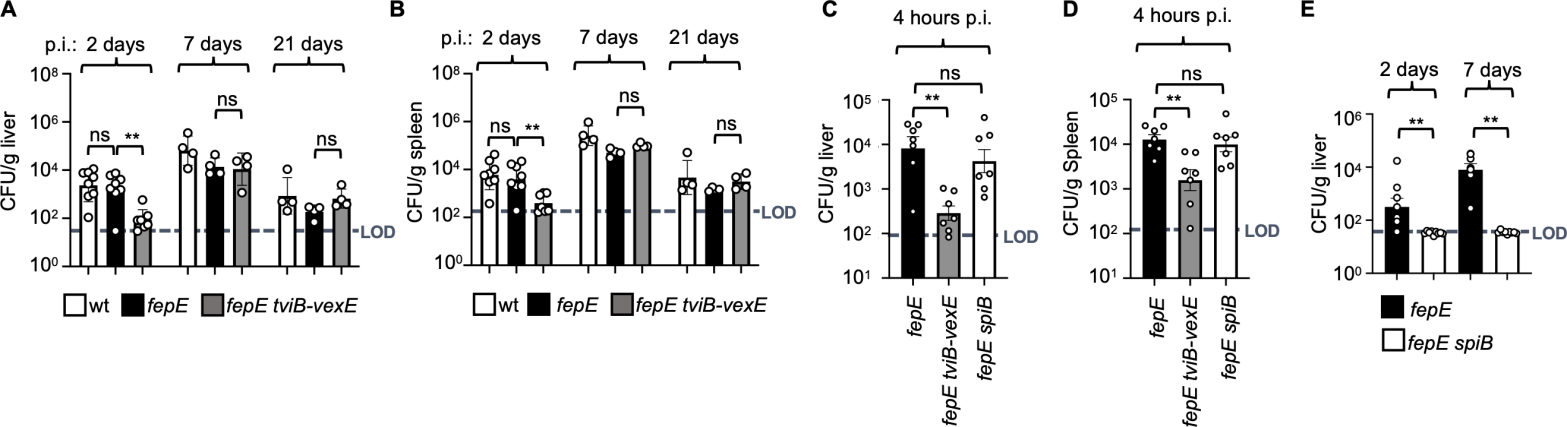
The Vi antigen enhances recovery of *S*. Paratyphi C from organs during the early stage of systemic infection. (**A, B, and E**) C57BL/6J mice were intraperitoneally infected with 10^4^ CFUs of *S*. Paratyphi C wild type (wt), *fepE* mutant, *fepE tviB-vexE* mutant, or *fepE spiB* mutant. (**C and D**) C57BL/6J mice were intraperitoneally infected with 10^6^ CFUs of the *S*. Paratyphi C isogenic mutant strains *fepE*, *fepE tviB-vexE*, or *fepE spiB*. The graphs depict the *Salmonella* CFUs recovered from the liver (**A, C, and E**) and spleen (**B and D**) at specified times post-infection. Each symbol represents data from an individual animal. LOD, limit of detection. Bars indicate geometric means ± standard error. Statistical significance is denoted as follows: ns, not significant, defined as *P* > 0.05; **, *P* < 0.01. The analysis was conducted using one-way analysis of variance followed by Tukey’s multiple comparison test (**A–D**) and Student’s *t*-test for comparisons between the *fepE* and *fepE spiB* groups (**E**).

To assess the role of the Vi antigen during systemic infection, 10^4^ CFUs of the *S*. Paratyphi C wild type, a *fepE* mutant, or a *fepE tviB-vexE* mutant were injected into the peritoneal cavity of mice. Two days after infection, the *fepE tviB-vexE* mutant was recovered in reduced numbers from the liver and spleen compared to the recovery of the parental *fepE* mutant (Fig. 3A and B). The *S*. Paratyphi C wild type and a *S*. Paratyphi C *fepE* mutant were recovered at similar numbers throughout the experiment (Fig. 3A and B). When *S*. Typhi is introduced into the murine abdominal cavity, it evades Vi antigen-dependent neutrophil phagocytosis as early as 1 h after inoculation (18). To model this interaction, we examined bacterial counts in the liver and spleen at very early stages after infection with a *S*. Paratyphi C *fepE* mutant or a *fepE tviB-vexE* mutant. Mice were infected intraperitoneally with 10^6^ CFUs to facilitate bacterial recovery 4 h later. Deletion of the Vi antigen biosynthesis genes resulted in reduced recovery of the pathogen from liver and spleen 4 h after infection (Fig. 3C and D). However, the Vi antigen was not required for bacterial recovery from the liver and spleen when mice were infected intragastrically with a *S*. Paratyphi C *fepE* mutant or a *fepE tviB-vexE* mutant (Fig. S1A through C).

### The SPI-2-encoded T3SS of *S*. Paratyphi C is required at later stages of infection

The T3SS encoded by SPI-2 (T3SS-2) is a well-characterized virulence factor that contributes to proliferation in macrophages as well as cytotoxicity in tissue culture and is required for growth of *S*. Typhimurium in murine tissue (46). Within T3SS-2, SpiB (SsaV) is an essential inner-membrane component of the export apparatus, forming the channel for effector secretion. It interacts with other core components, including the ATPase SsaN and the C-ring protein SsaQ, coordinating substrate recognition and effector delivery (47). Deletion of *spiB* abolishes T3SS-2 function, preventing effector translocation and markedly attenuating *Salmonella* virulence in both macrophages and animal models. These findings highlight SpiB as indispensable for the structural integrity and activity of the SPI-2 secretion system. To assess T3SS-2 function in a *S*. Typhi-like surface context, the *fepE* mutant of *S*. Paratyphi C was used, and *spiB* was subsequently deleted, enabling evaluation of T3SS-2 activity while maintaining an outer membrane architecture comparable to that of *S*. Typhi. Inactivation of T3SS-2 by deleting the *spiB* gene in the *S*. Paratyphi C *fepE* mutant reduced cytotoxicity and proliferation in murine-like RAW264.7 macrophages (Fig. S2A through D). Inactivation of T3SS-2 did not reduce bacterial recovery 4 h after intraperitoneal infection (Fig. 3C and D) but markedly decreased bacterial recovery 2 days after infection (Fig. 3E; Fig. S2E). These results suggest that the Vi antigen and T3SS-2 are required during the early or later phases of *S*. Paratyphi C infection, respectively.

### *S*. Paratyphi C infection of mice can be used to model vaccine-mediated protection

**Fig 4:**
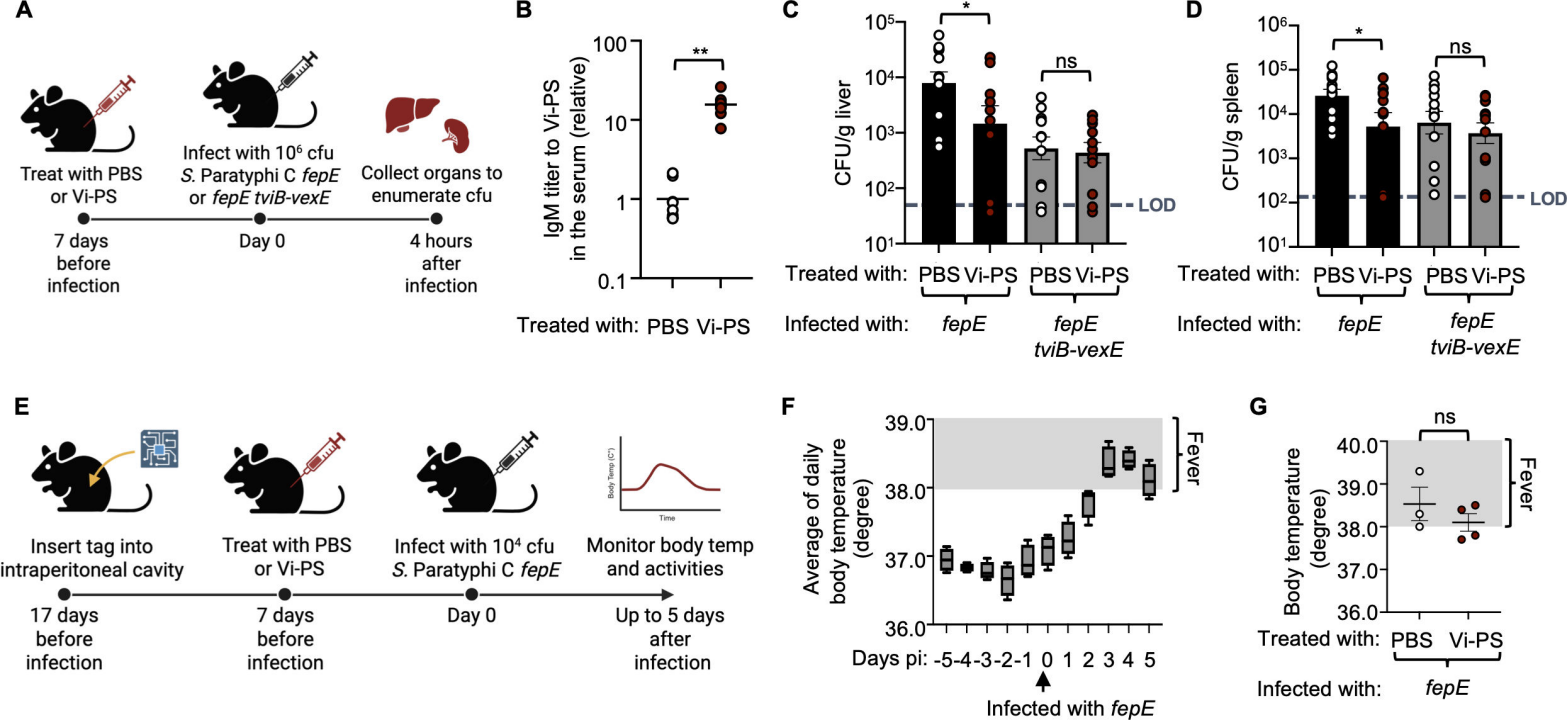
*S*. Paratyphi C infection of mice can be used to model vaccine-mediated protection. (**A**) Diagram of the experimental groups and time points. Vi-PS denotes the purified Vi antigen. (**B**) Comparative analysis of the relative IgM titer in the serum of mice administered Vi-PS in contrast to those treated with PBS. The analysis was conducted using the Mann-Whitney test. (**C and D**) Seven days post-treatment with either PBS or Vi-PS, C57BL/6J mice were intraperitoneally infected with 10^6^ CFUs of *S*. Paratyphi C *fepE* or *fepE tviB-vexE* mutant. The graphs depict the *Salmonella* CFUs recovered from the liver (**C**) and spleen (**D**) at 4 h post-infection (pi.). The analysis was conducted using Student’s *t*-test. (**E**) Diagram of the experimental groups and time points. Body temperature of mice installed with a tag was monitored by a novel biotelemetry system, eeeHive SENS. (**F**) Transition of average of daily body temperature of mice (*n* = 4) 5 days before and after 10^4^ CFUs of *S*. Paratyphi C *fepE* mutant infection. (**G**) Average of daily body temperature of mice treated with PBS or Vi-PS 3 days after 10^4^ CFUs of *S*. Paratyphi C *fepE* mutant infection. The analysis was conducted using Student’s *t*-test. Each symbol represents data from an individual animal. Bars indicate geometric means ± standard error. Statistical significance is denoted as follows: ns, not significant, defined as *P* > 0.05, *P < 0.05, and **P < 0.01.

For vaccine development, it is important to have access to an animal model that can be used to establish a proof of concept for protection (24). Therefore, to assess the potential of the mouse model of *S*. Paratyphi C infection for vaccine development, we inoculated mice intraperitoneally with either phosphate-buffered saline (PBS) or purified Vi antigen (Vi-PS) and evaluated their resistance to subsequent infection with the capsulated *S*. Paratyphi C *fepE* mutant or a non-capsulated *S*. Paratyphi C *fepE tviB-vexE* mutant (Fig. 4A). Seven days after Vi-PS administration, the level of IgM to Vi-PS in mouse serum was significantly elevated compared to that in mice inoculated with PBS (Fig. 4B). Four hours after intraperitoneal inoculation with 10^6^ CFUs of capsulated *S*. Paratyphi C (*fepE* mutant), the bacterial count in the liver and spleen of mice immunized with Vi-PS was significantly lower than that in the PBS-treated group (Fig. 4C and D). However, no significant difference was observed between the two groups after 4 days of infection (Fig. S3A and B). In contrast, immunization with Vi-PS did not lower bacterial counts in mice infected with a non-capsulated *S*. Paratyphi C *fepE tviB-vexE* mutant (Fig. 4C and D). This observation suggests that the IgM levels to Vi-PS are important for vaccine protection in the acute stage of *S*. Typhi infection, consistent with previous findings (48, 49).

### Mice infected with *S*. Paratyphi C develop a febrile illness

Humans infected with *S*. Typhimurium develop diarrhea, vomiting, intestinal cramping, and fever less than 24 h after ingestion (50). In contrast, individuals infected with *S*. Typhi develop fever as the first symptom after a prolonged incubation period of several days to 2 weeks (4). To determine whether *S*. Paratyphi C infection of mice produces signs of disease that resemble symptoms of typhoid fever, we wanted to monitor the body temperature of mice during *S*. Paratyphi C infection. Since skin temperature of mice can differ from the core body temperature, we implanted a biotelemetry device, eeeHive SENS, developed at Phenovance LCC (Fig. S3C). Prior to infection, the biotelemetry system accurately detected fluctuations in body temperature consistent with the known circadian rhythm of the mouse core body temperature, with daily averages staying below 37°C (Fig. 4F; Fig. S3D). Parenteral infection with 10^4^ CFUs of a *S*. Paratyphi C *fepE* mutant induced fever exceeding a daily average of 38°C starting on day 3 days after infection (Fig. 4F). Activity recorded with the biotelemetry device was negatively correlated with the body temperature (Fig. S3E and F). Vaccination with Vi-PS did not significantly suppress the rise in body temperature observed during *S*. Paratyphi C infection (Fig. 4C, D and G). Collectively, these results suggest that the use of *S*. Paratyphi C as a surrogate pathogen to study typhoid fever pathogenesis in the mouse reproduces symptoms observed in humans, including the development of fever after a prolonged incubation period.

## DISCUSSION

Similar to *S*. Typhi, *S*. Paratyphi C produces the Vi antigen. However, unlike *S*. Typhi, it shares an evolutionary ancestor with *S*. Chorelaesuis and *S*. Typhisuis, which are pathogens that infect pigs (51, 52). Approximately 4,000 years ago, the *S*. Paratyphi C clade diverged from a common ancestor shared with *S*. Chorelaesuis through the acquisition of genes, such as the *viaB* locus, by horizontal gene transfer (43) and the accumulation of pseudogenes (52). *S*. Paratyphi C produces less Vi antigen than *S*. Typhi and *Citrobacter freundii* (44); however, our data suggest that the *S*. Paratyphi C Vi antigen reduced ROS production by neutrophils *in vitro* to a similar degree as the *S*. Typhi Vi antigen, and differences in the interaction of these serovars with neutrophils were attributable to the absence of VL O-antigen chains in the *S*. Typhi. Clinical isolates of *S*. Typhi can carry mutations in *tviE* that result in heightened (hyper Vi variant) or decreased (hypo Vi variant) synthesis of the Vi antigen, which increases or lowers virulence in a mouse infection model, respectively (53). The *S*. Paratyphi C strain 13428 carries a *tviE* K266N mutation, which results in a hypo Vi variant in *S*. Typhi (53). Therefore, deleting *fepE* in *S*. Paratyphi C provides a model for characterizing the virulence of hypo or hyper Vi variants generated by introducing point mutations in *tviE* found in clinical *S*. Typhi isolates.

TviA, a transcriptional regulator of Vi antigen biosynthesis that also represses SPI-1 and flagellar genes, is subject to temperature-dependent regulation via its 5′ UTR in *S*. Typhi (54). In S. Paratyphi C, the tviA 5′ UTR harbors three nucleotide substitutions and lacks a bulging adenine immediately upstream of the Shine-Dalgarno region, potentially altering RNA thermosensor melting and modulating *tviA* translation. These features may affect the timing and level of Vi antigen expression upon entry into host tissues. Together with the *tviE* K266N mutation, which produces shorter and less abundant Vi antigen (53), these factors likely account for the absence of differences in bacterial recovery following intragastric infection, whereas intraperitoneal infection exposes bacteria directly to neutrophils, revealing a clear Vi-dependent advantage.

Because *S*. Typhi is not transmissible to other animals, the carrier plays a crucial role in the transmission of typhoid infection. In 1%–5% of individuals infected with typhoid, *S*. Typhi chronically infects the gallbladder and subsequently passes through the bile duct into the intestinal tract (55–57). Apparently healthy individuals who chronically shed *S*. Typhi serve as an important reservoir for human-to-human transmission of the pathogen (58). Approximately 90% of carriers also present gallstones, in which *Salmonella* forms biofilms (59, 60). An association between chronic bacterial carriage and gallbladder cancer has been suggested (61–63), and the flagellar gene for *S*. Typhi was detected in 67.3% of gallbladder cancer patients in North India, a significantly higher prevalence than that in the control group (64). Analyzing the mechanisms underlying such chronic infections and developing treatments are critical for the control of typhoid fever. *S*. Typhimurium has been reported to colonize gallstones in a murine infection model in mice fed a lithogenic diet (59, 60). Similarly, identifying factors that promote colonization of *S*. Paratyphi C in the gallbladder will facilitate the development of a typhoid model capable of analyzing both acute and chronic infections.

Typhoidal *Salmonella* infection predominantly affects individuals aged 1–15 years, resulting in a higher number of years of life lost compared to the mortality rate (7). However, existing typhoid vaccines, such as Ty21a and Vi-PS, are unsuitable for children under 2 years of age and exhibit limited efficacy (20). Therefore, there is a pressing need to develop conjugate vaccines targeting the Vi antigen, which are both highly effective and durable in young children, such as Vi-rEPA and the TCV (20). The recent surge in cases of XDR *S*. Typhi infection has also emerged as a significant concern (6, 65, 66). The WHO is advising that addressing the problem of drug-resistant typhoidal *Salmonella* serovars is of high priority for public health (8). Consequently, it is imperative to explore diverse treatment strategies, such as the identification of novel antimicrobial compounds, phage therapy, or combinations thereof (67). Additionally, the application of deep learning and artificial intelligence in the design of antimicrobial agents has been investigated, with the potential for efficient drug development through innovative technologies (68–70). In this context, the use of *S*. Paratyphi C as a surrogate pathogen to study typhoid fever pathogenesis using the mouse model is a valuable addition for evaluating the efficacy of future vaccines and therapeutics.

## MATERIALS AND METHODS

### Bacterial strains and culture conditions

The bacterial strains and plasmids used in the present study are listed in Table S1. Some isogenic mutant strains and plasmids used in this study were constructed using primers listed in Table S1 as described previously (19). *Salmonella* strains were routinely cultured at 37°C with aeration in Luria-Bertani (LB) broth (10 g tryptone, 5 g yeast extract, and 10 g NaCl per liter) or on LB agar plates, unless stated otherwise. Antibiotics were added to the medium when required. To stimulate expression of Vi antigen, bacterial strains were grown in LB broth with reduced sodium concentration (0.5% NaCl).

### Animals

Female 7- to 8-week-old C57BL/6J mice were purchased from CLEA Japan, Japan SLC, or Jackson Laboratories (stock no. 000664). Upon arrival, mice from each cohort were randomly assigned to individually ventilated cages on one rack at a housing density of 3–4 animals per cage and allowed to acclimate in the vivarium for at least a week. Undisturbed 70% ethanol was used to disinfect the surfaces and gloves between groups. The number of animals used in each group is indicated in each graph or the figure legend.

### *Salmonella* infection of mice

*Salmonella* strains were grown overnight, and 10^4^ or 10^6^ CFUs of the indicated strains suspended in 100 µL PBS were injected intraperitoneally into C57BL/6J mice. For intragastric infection, 100 µL of 10^10^ CFUs/mL bacterial suspension in LB broth was orally administered using a Sonde needle. Indicated days after infection, the CFU of each strain was determined in homogenates of the liver, spleen, colon content, and feces by spreading serial 10-fold dilutions on agar plates containing the appropriate antibiotics, and *Salmonella* CFUs per gram of tissues were calculated. For enumeration of bacterial numbers in feces, the mouse cage was changed every single day. Spleen weight was measured to assess splenomegaly prior to homogenization.

### Vi-PS vaccination of mice

The purified Vi capsule polysaccharide (Vi-PS) used for vaccination was purified as described for capsular polysaccharides from *Bacteroides fragilis* (71). C57BL/6J mice were intraperitoneally immunized with 100 µL of Vi-PS (2 µg) or PBS as mock treatment. Seven days after immunization, to measure the titer of anti-Vi-PS immunoglobulin, sera were obtained from the collected whole blood. On the same day, to see the efficacy of the Vi-PS vaccination against *Salmonella* strains expressing Vi antigen, 10^6^ CFUs of *S*. Paratyphi C *fepE* and *fepE tviB-vexE* mutant strains were intraperitoneally infected, and CFUs in the liver and spleen after the indicated days were enumerated as described above.

### Monitoring body temperature and activity of infected animals

To monitor the body temperature and body movement of mice during infection, we used a wireless-powered biotelemetry system, eeeHive SENS, developed at Phenovance LCC (Chiba, Japan; Fig. S3C). This biotelemetry uses an implantable measurement tag incorporating a coil antenna on a flexible printed circuit, a temperature (accuracy ±0.1°C), and a six-axis inertial sensor (three-axis accelerometer and three-axis gyroscope). Ten days after the tag was sterilely installed under anesthesia with medetomidine-midazolam-butorphanol, mice were treated with 100 µL of Vi-PS (2 µg) or PBS as mock treatment, and 10^4^ CFUs of *S*. Paratyphi C *fepE* mutant were challenged 7 days after the treatment described as above. We analyzed murine body temperatures (degree) and activity (three-axis acceleration) and graphed data from 5 days prior to and following infection. The biotelemetry system could accurately demonstrate that body temperature variations adhere to circadian and crepuscular rhythms, with an increase observed post-infection (Fig. S3D).

### Enzyme-linked immunosorbent assay

The procedure for measuring Vi-PS-specific IgM was adapted from the assay described by Rigsby et al. (72). In brief, F8 Maxisorp Loose Nunc-Immunu Module strips (Thermo Fisher Scientific, #469949) were pre-coated with 100 μL of poly L-lysine hydrobromide (Cosmo Bio Co, SPL01 100ML) at a concentration of 10 mg/mL and incubated for 2 h at room temperature (RT). Subsequently, 100 μL of Vi-PS (5 μg/mL) was added, and the mixture was incubated overnight at 37°C. The plates were blocked with 1% bovine serum albumin (BSA) in PBS for 1 h at 37°C. Serum samples were serially diluted in PBS-T (PBS containing 0.25% Tween-20) with 1% BSA, starting at 1:50 for IgM, then 100 μL was added to each well. The plates were then incubated for 2 h at 37°C. Bound antibodies were detected using alkaline phosphatase-conjugated goat anti-mouse Ig secondary antibodies (Southern Biotech), diluted 1:4,000 for IgM (#1021-04). Detection was performed using p-nitrophenyl phosphate substrate (Sigma-Aldrich). After 1 h of incubation, the absorbance at 405 nm was measured using a Varioskan LUX Multimode Microplate Reader (Thermo Fisher Scientific). The relative titer of samples over the PBS (mock) immunized control was determined based on the dilution at which a defined threshold was reached.

### Slide agglutination test

Harvested bacterial pellets from 500 µL of *Salmonella* overnight culture were resuspended in a small volume of PBS (50 µL). Ten microliter of the condensed bacterial suspension and 10 µL anti-O4 (Denka Seiken, #211262), -O7 (Denka Seiken, #211279), and -Vi (Denka Seiken, #211408) anti-rabbit sera were mixed and incubated for 5 min at RT. Aggregated bacterial clumps were monitored and scored as negative (−), positive (+), or robust (+++). Anti-O4, which binds the LPS of *S*. Typhimurium, was included as a negative control in assays with *S*. Paratyphi C.

### LPS silver stain analysis

LPS from *S*. Paratyphi C strains was purified from overnight cultures using an LPS extraction kit (iNtRON Biotechnology). A sensitive silver staining protocol (73) was then used to detect purified LPS, which was separated by SDS-PAGE.

### ROS production assay

ROS production by murine neutrophils was assessed using a luminol-based chemiluminescence assay, as described previously (19). Primary murine neutrophils were isolated from C57BL/6J mice following euthanasia. Bone marrow cells were harvested, and neutrophils were enriched using the EasySep Mouse Neutrophil Enrichment Kit (StemCell Technologies) in accordance with the manufacturer’s instructions. Murine neutrophils were washed and resuspended in phenol red-free RPMI 1640 supplemented with 2% fetal bovine serum and 1 mM luminol (Sigma-Aldrich). Approximately 5 × 10⁴ neutrophils (90 μL) were seeded into each well of an opaque 96-well microplate. Baseline luminescence was recorded every 2 min for 5 min using a Varioskan LUX Multimode Microplate Reader (Thermo Fisher Scientific). Subsequently, 10 μL of bacteria (multiplicity of infection of 10), either opsonized with 20% murine serum (incubated at RT for 25 min) or mock-treated, was added to each well. ROS production was monitored every 2 min using a microplate reader.

### Dot blot assay

LPS and Vi antigens were purified from *Salmonella* overnight cultures using an LPS extraction kit (iNtRON Biotechnology). A volume of 5 μL of each sample was spotted onto a nitrocellulose membrane and air-dried. Membranes were blocked with 5% skim milk in PBS-T. To detect specific O or Vi antigens, anti-O7 (Denka Seiken, #211279) and anti-Vi (BD Biosciences, #228271) anti-rabbit sera were used. A secondary horseradish peroxidase-conjugated anti-rabbit IgG antibody (Invitrogen, #61-6520) was used to detect bound primary antibodies. Detection was performed using enhanced chemiluminescence (ECL; Amersham Biosciences), and images were acquired using a LuminoGraph II System (Atto).

### Statistical analysis

All data are presented as the mean geometric means ± standard error of the mean. GraphPad Prism v10 was used for statistical analyses, and the test used to analyze each data set is indicated in the figure legend. *P* < 0.05 was considered significant.

## Data Availability

All raw data are available in the supplemental material.

## SUPPLEMENTAL MATERIAL

Supplemental Material mbio.03622-25-s0001.docx Supplemental text, table, and figures.

Raw Data mbio.03622-25-s0002.xlsx Raw data for figures.

ASM does not own the copyrights to Supplemental Material that may be linked to, or accessed through, an article. The authors have granted ASM a non-exclusive, world-wide license to publish the Supplemental Material files. Please contact the corresponding author directly for reuse.
