# Supplementary material for: A new mouse model of typhoid fever using Salmonella enterica serovar Paratyphi C as a surrogate pathogen
Source: mBio. 2026 Jan 16;17(2):e03622-25. doi: 10.1128/mbio.03622-25 (PMC12892999; doi:10.1128/mbio.03622-25)
Supplement: Supplemental Material — Supplemental text, table, and figures. [file mbio.03622-25-s0001.docx]

**SUPPLEMENTAL MATERIAL**

**A new mouse model of typhoid fever using *Salmonella enterica* serovar Paratyphi C as a surrogate pathogen**

Hoan T. Pham^1,2^, Masatomo Morita^3^, Kohei Yamazaki^4^, Toshihiro Endo^5^, Satoshi Takayama^1^, Azusa Hiyoshi^1^, Takeshi Haneda^6^, Renée M. Tsolis^7^, Andreas J. Bäumler^7^, Toshio Kodama^1,$*^, Hirotaka Hiyoshi^1,$*^

**SUPPLEMENTAL MATERIALS AND METHODS**

***Gentamicin protection assay***

To determine intracellular bacterial proliferation in phagocytes, RAW264.7 murine macrophage-like cells were infected with *S.* Paratyphi C strains. Cells were seeded in 24-well plates at a density of 2 × 10^5^ cells/well. Bacteria were added to cells at a multiplicity of infection (MOI) of 10 bacteria per cell. The plate was centrifuged at 250 g for 5 min and incubated (37°C and 5% CO2) for 30 min. After this incubation, RPMI medium (0.5 mL) containing 0.1 mg/mL gentamicin (Gibco) was then added to the cells for 30 min at 37°C to kill extracellular bacteria. After incubation, the concentration of gentamicin was reduced to 10 µg/mL and cells until lysed for enumerating cfu in RAW264.7 cells until lysed to enumerating CFU in RAW264.7 cells. 3 hours and 21 hours after incubation with RPMI medium containing gentamicin, cells were washed three times with PBS (0.5 mL) and then lysed with pre-chilled sterile water (0.5 mL) for 15 minutes. The recovery of bacteria from phagocytes was quantified by spreading serial 10-fold dilutions on LB agar plates with appropriate antibiotics to enumerate the CFUs.

***Cytotoxicity***

To investigate the cytotoxic activity of *S*. Paratyphi C in RAW264.7 cells, time-lapse live-cell imaging was conducted using the Sartorius Incucyte SX1 system. The cells were seeded in 96-well plates (ibidi, #ib89626) at a density of 1 × 10^5^ cells/well. Bacteria were added to cells at a multiplicity of infection (MOI) of 100 bacteria per cell. The plate was centrifuged at 250 g for 5 min and incubated (37°C and 5% CO2) for 30 min. After this incubation, RPMI medium (0.3 mL) containing 0.1 mg/mL gentamicin (Gibco) was then added to the cells for 30 min at 37°C to kill extracellular bacteria. After incubation, the concentration of gentamicin was reduced to 10 µg/mL, and the 96-well plate was transferred to the Incucyte SX1 system. Images were captured at 20× magnification at specified time intervals, with three–four fields of view per well. The cells were stained with SYTOX Green (0.5 µM, Invitrogen, #S7020). The Incucyte Cell-By-Cell Analysis Module segmented individual cells and identified dead cells based on fluorescence intensity and morphology. Cytotoxicity was quantified by calculating the percentage of dead cells relative to the total cell coverage, estimated from phase-contrast confluence.

**SUPPLEMENTAL TABLE**

**Table S1: Bacterial strains, plasmids, and primers for qPCR used in this study.**

| Strain designation | Relevant characteristics/Genotype | Source/Reference |
| --- | --- | --- |
| *S*. Typhimurium |  |  |
| IR715 | ATCC14028 Nal^R^, wild type | (1) |
| *S.* Typhi |  |  |
| Ty2 | ATCC700931, wild-type strain | ATCC |
| *S*. Paratyphi C |  |  |
| ATCC13428 | 13428, wild type | ATCC |
| HP8 | 13428 *fepE*, very-long O antigen deficient by deletion of *fepE* gene. | This study |
| HH429 | 13428 *tviB-vexE*, Vi antigen deficient strain by deletion of *tviB-vexE* locus. | This study |
| HP11 | 13428 *fepE tviB-vexE*, very-long O and Vi antigens deficient strain by deletion of *fepE* gene and *tviB-vexE* locus. | This study |
| HP27 | 13428 *fepE spiB*, very-long O antigen and T3SS-2 deficient strain by deletion of *fepE* and *spiB* genes. | This study |
| HP122 | 13428 *fepE invA*, very-long O antigen and T3SS-1 deficient strain by deletion of *fepE* and *invA* genes. | This study |
| HP129 | 13428 *fepE invA spiB*, very-long O antigen, T3SS-1 and T3SS-2 deficient strain by deletion of *fepE, invA* and *spiB* genes. | This study |
| Plasmid |  |  |
| pAmp^r^-30C | Truncated pKD46, temperature-sensitive plasmid conferring carbenicillin resistance under 30 degrees. | This study |
| *pfepE* | *fepE* from 13428 in pWSK29 | This study |
| *pspiB* | *spiB* from 13428 in pWSK29 | This study |
| Primer |  |  |
| *∆fepE*-1 | GGATCCGGTGCCGGAAGGCGTATCG | This study |
| *∆fepE*-2 | GGTAAATCAGACTAACCGTTAAGAGATGGCATGCCGTATGACC | This study |
| *∆fepE*-3 | GGTCATACGGCATGCCATCTCTTAACGGTTAGTCTGATTTACC | This study |
| *∆fepE*-4 | GTCGACAGGTATCACCTCACTGGCGG | This study |
| *∆tviB-vexE*-1 | GTCGACAGCATAGGAGACTTCATGAGG | This study |
| *∆tviB-vexE*-2 | GTAGCGTATCTGAATGTCACCATCAGTAGCACAACG | This study |
| *∆tviB-vexE*-3 | CGTTGTGCTACTGATGGTGACATTCAGATACGCTAC | This study |
| *∆tviB-vexE*-4 | GTCGACGAGCAGAAGAGCTCGGTGTCAG | This study |
| *∆spiB*-1 | GTCGACGCGTTACACCTCGCTTG | This study |
| *∆spiB*-2 | GTTGTCATTTTCCACTCACGGATTAACCATGAGATATGCC | This study |
| *∆spiB*-3 | GGCATATCTCATGGTTAATCCGTGAGTGGAAAATGACAAC | This study |
| *∆spiB*-4 | AGATCTGGCTGCGCCAATTAACGCCTGG | This study |
| *∆invA*-1 | GGATCCGGGTTCGCTATTAACCGATA | This study |
| *∆invA*-2 | TAATTAAGCCCTTATATTGTGAAAGCAGCACTATAGGTATCCTG | This study |
| *∆invA*-3 | CAGGATACCTATAGTGCTGCTTTCACAATATAAGGGCTTAATTA | This study |
| *∆invA*-4 | GTCGACGGGTAGGCCAGATATTGCAG | This study |
| *fepE*-Fw | GTCGACCACTGGCGCGTAAAGATTG | This study |
| *fepE*-Rv | GGATCCTCAGACTAACCGTTCATCTATCG | This study |
| *spiB*-Fw | GGATCCGGGCGATATCCCTGTCGTAG | This study |
| *spiB*-Rv | GTCGACCCACTCACTTAAAATCTAATGG | This study |

**SUPPLEMENTAL FIGURES**


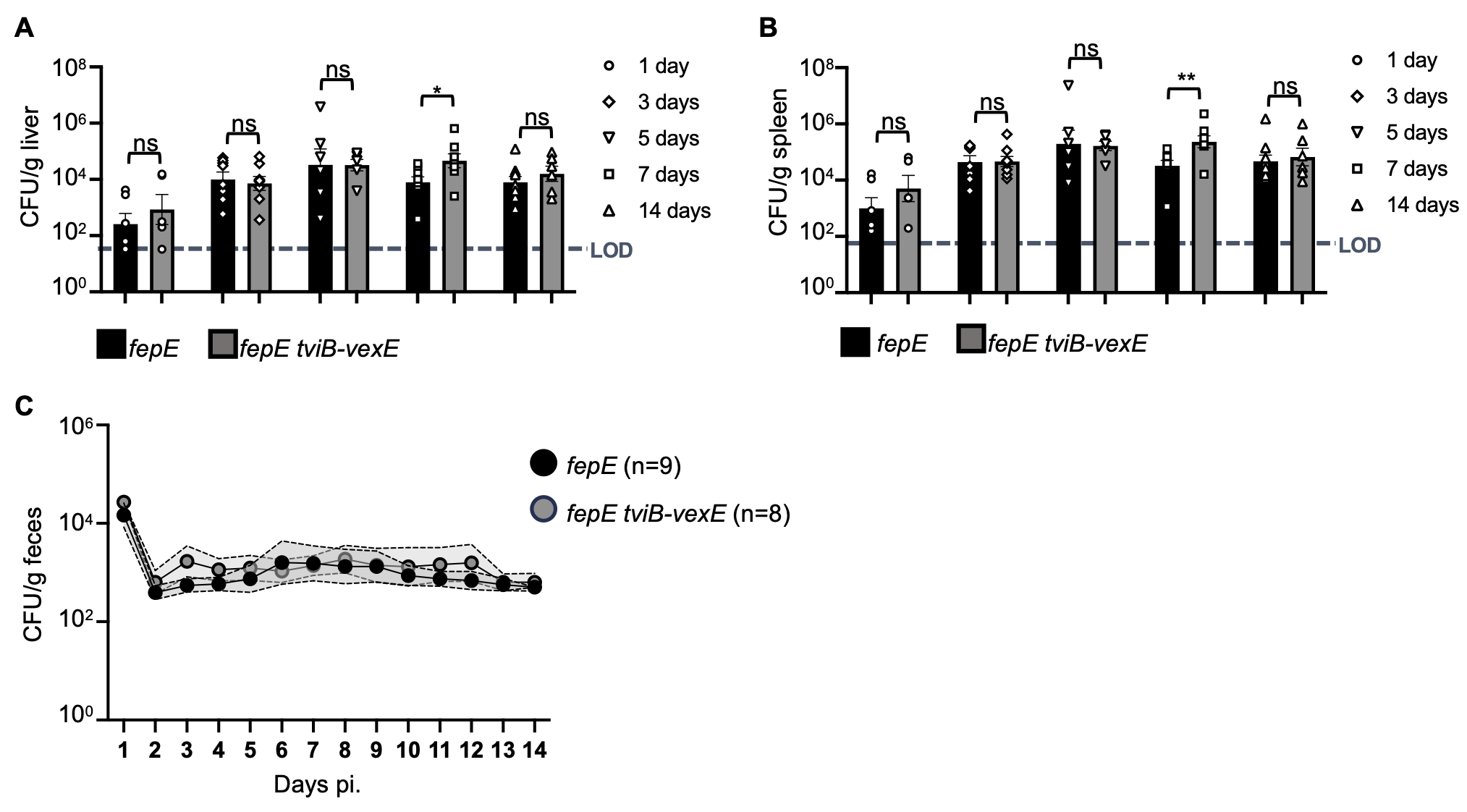


**Supplemental fig. 1:** **Investigation of the role of Vi antigen in *S*. Paratyphi C systemic infection via the oral infection route.**

C57BL/6J mice were orally infected with 10^9^ CFUs of the *S*. Paratyphi C 13428 isogenic mutant strains *fepE* and *fepE tviB-vexE*. The graphs illustrate the *Salmonella* CFUs recovered from the liver (A), spleen (B), and feces (C) on specific days post-infection. Each symbol represents data from an individual animal. LOD, limit of detection. Bars indicate geometric means ± standard error. Statistical significance is denoted as follows: ns, not significant, defined as p>0.05; *, p<0.05; **, p<0.01. The analysis was conducted using Student’s t-test.


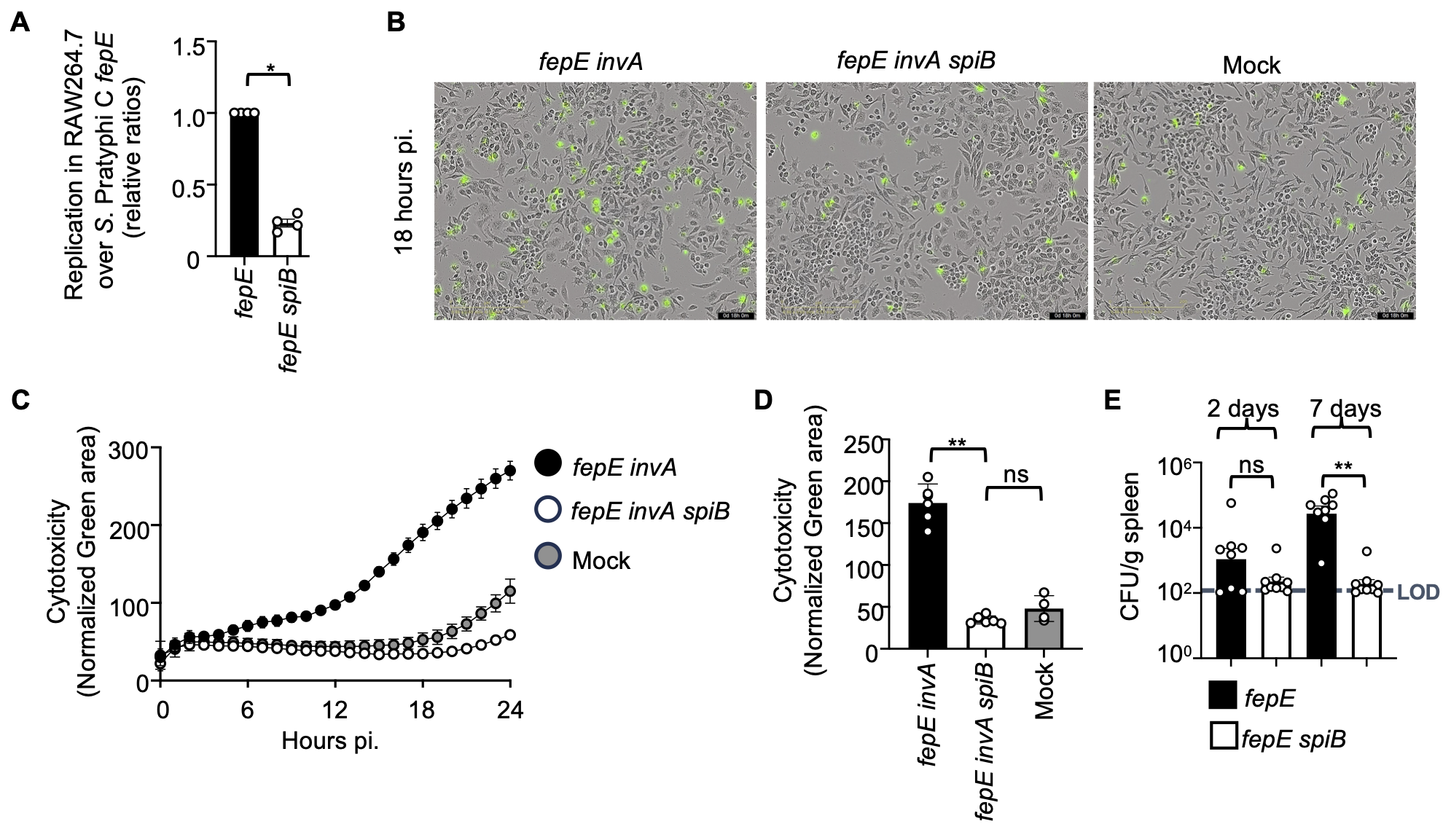


**Supplemental fig. 2:** **The role of T3SS-2 in *S*. Paratyphi C is analogous to that in *S*. Typhimurium.**

(A) RAW264.7, murine macrophage-like cells were infected with *S*. Paratyphi C isogenic mutant strains *fepE* and *fepE spiB*. The replication rates from 3 hours to 21 hours after infection were measured for each strain, and the differences compared to *fepE* mutant are shown as relative ratios. The analysis was conducted using Student’s t-test for the two groups. (B) Dead cells infected with *S*. Paratyphi C strains or remained mock infection after 18 hours post infection (pi.) were stained with SYTOX Green. (C) Cytotoxicity, measured by normalizing cell area stained with SYTOX Green by that of total RAW264.7 cells in a well, is plotted for each hour. (D) Cytotoxicity infected with *S*. Paratyphi C strains or remained mock infection after 18 hours pi. were assessed repeatedly and potted on the graph. The analysis was conducted using one-way ANOVA followed by Tukey’s multiple comparison test. (E) C57BL/6J mice were intraperitoneally infected with 10^4^ colony-forming units (CFUs) of *S*. Paratyphi C *fepE* and *fepE spiB* mutants. The graphs depict the *Salmonella* CFUs recovered from the liver at the specified times post-infection. Each symbol represents data from an individual animal. LOD, limit of detection. Bars indicate geometric means ± standard error. Statistical significance is denoted as follows: ns, not significant, defined as p>0.05; **, p<0.01. significance is denoted as follows: ns, not significant, defined as p>0.05; **, p<0.01. The analysis was performed using Student’s t-test for the two groups.


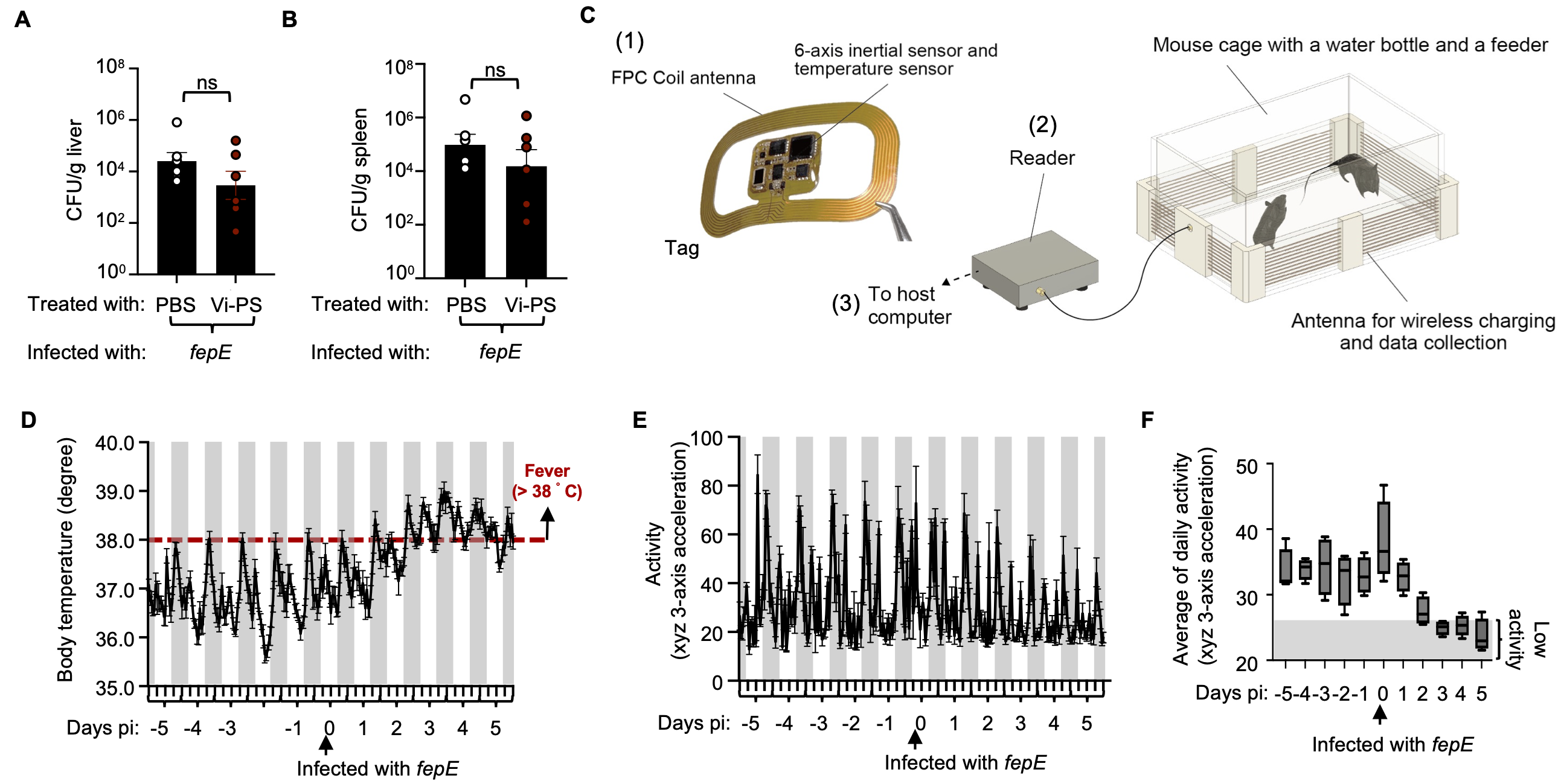


**Supplemental fig. 3:** **A novel biotelemetry, eeeHive SENS, revealed step-by-step alteration in body temperature of mice infected with *S*. Paratyphi C.**

(A, B) 7 days post-treatment with either PBS or Vi-PS, C57BL/6J mice were intraperitoneally infected with 10^4^ colony-forming units (CFUs) of *S*. Paratyphi C *fepE*. The graphs depict the *Salmonella* CFUs recovered from the liver (A) and spleen (B) at 4 days post-infection. The analysis was conducted using Student’s t-test. (C) Diagram illustrating the composition of the novel biotelemetry system, eeeHive SENS. The eeeHive SENS consisted of three components: (1) an implantable measurement tag (Tag) incorporating a coil antenna on a flexible printed circuit (FPC), a temperature sensor (accuracy ±0.1 °C), and a 6-axis inertial sensor (3-axis accelerometer and 3-axis gyroscope); (2) a reader unit and reader that enabled simultaneous wireless power transfer and data acquisition from multiple tags; and (3) a host computer. (D, E) Average hourly body temperature (D) and activity (E) of mice infected with 10^4^ CFUs of *S*. Paratyphi C *fepE* mutant (n=4). Gray bars indicate the period between dawn and dusk. (F) Transition of average of daily activity of mice (n=4) 5 days before and after 10^4^ CFUs of *S*. Paratyphi C *fepE* mutant infection. Bars indicate geometric means ± standard error. Statistical significance is denoted as follows: ns, not significant, defined as p>0.05.

**SUPPLEMENTAL REFERENCE**

1. Stojiljkovic I, Bäumler AJ, Heffron F. 1995. Ethanolamine utilization in Salmonella typhimurium: nucleotide sequence, protein expression, and mutational analysis of the cchA cchB eutE eutJ eutG eutH gene cluster. J Bacteriol 177:1357-66.
